# An inverted U-shaped relationship between chronic stress and the motivation to expend effort for reward

**DOI:** 10.1016/j.ynstr.2025.100724

**Published:** 2025-04-05

**Authors:** Wei Yi, Xin Li, Wangxiao Chen, Linlin Yan, Fei Xin, Tony W. Buchanan, Jianhui Wu

**Affiliations:** a School of Psychology, Shenzhen University, Shenzhen, China; b Donders Institute for Brain, Cognition and Behavior, Radboud University Medical Center, Nijmegen, the Netherlands; c Department of Psychology, Saint Louis University, St. Louis, MO, USA

**Keywords:** Chronic stress, Motivation, Effort, RewP, Drift-diffusion model

## Abstract

Dysfunction in the motivation to expend effort for reward is considered a crucial symptom of stress-related mental illness. Few studies have explored the relationship between chronic stress and the motivation to exert effort for reward, along with its underlying neural mechanisms. We investigated this relationship in ninety undergraduates who were undergoing a chronic stressor: preparing for the National Postgraduate Entrance Examination (NPEE). Students engaged in an effort-reward task while EEG signals were recorded, wherein they could accept or reject an offer to expend effort for another opportunity to obtain the reward. Participants’ chronic stress levels were assessed using the Perceived Stress Scale (PSS) and their decision was further captured by a drift-diffusion model (DDM). Compared to reward omission, reward delivery led to increased amplitude of the reward positivity (RewP) ERP waveform, particularly in extra reward trials relative to regular trials. Importantly, the PSS score showed an inverted U-shaped relationship with the motivation indicators, including offer acceptance rate (behavioral index), drift rate (model parameter), and ΔRewP (i.e., the difference in RewP in response to reward delivery compared to reward omission, ERP component). These findings suggest an inverted U-shaped relationship between chronic stress and motivation, suggesting that individuals display diminished motivation when exposed to low or high levels, relative to moderate levels, of chronic stress. Our study holds significant implications for understanding both vulnerability and resilience to stress-related mental disorders.

## 1 Introduction

The motivation to exert effort for rewards is essential for survival (M. Inzlicht, A. Shenhav, & C. Y. Olivola, 2018; Kurzban, 2016). Expending effort is costly, requiring continual cognitive control for adjustment depending on the context. According to the expected value of control (EVC) model, people adjust their effort expenditure according to the potential gains in a given context (Shenhav et al., 2013; Shenhav et al., 2017; Shenhav et al., 2021). The motivation to allocate effort is associated with activity in a network including the prefrontal cortex (PFC), ventral striatum (VS), and amygdala (Botvinick and Braver, 2015; Cunningham and Brosch, 2012; Ott and Nieder, 2019; Shenhav et al., 2021; Warlow and Berridge, 2021). Additionally, dopamine plays a crucial role in motivating individuals to exert cognitive effort, and mediating decision-making about effortful action (Cools, 2016; Cools et al., 2019; Ott and Nieder, 2019; Salamone et al., 2018; Westbrook and Braver, 2016). Previous studies have shown that dopamine enhances individuals’ willingness to exert effort for rewards, possibly by altering the cost-benefit balance of cognitive tasks (McGuigan et al., 2019; Westbrook et al., 2020). Dysfunction of motivation to allocate effort is associated with diverse mental diseases, including major depressive disorder (MDD; (Admon and Pizzagalli, 2015; Grahek et al., 2019), schizophrenia (Blouzard et al., 2023; Catalano and Green, 2023; Culbreth et al., 2018; Medalia and Brekke, 2010; Wolf et al., 2014), and PTSD (Ben-Zion et al., 2022; Nawijn et al., 2015).

While chronic stress is recognized as a risk factor for various motivation-related mental disorders (Belleau et al., 2019; Davis et al., 2017; Rohleder, 2016; Sheline et al., 2019; Starcke and Brand, 2012), the relationship between chronic stress and the motivation to allocate effort remains unclear. Evidence from animal models suggests that chronic stress impairs effort-related choice behaviors (Cerniauskas et al., 2019; Kleen et al., 2006; Rygula et al., 2005; Yoshida et al., 2021) by influencing the function of motivation-related brain regions and dopamine levels (Cerniauskas et al., 2019; Hollon et al., 2015; Wook Koo et al., 2016; Yoshida et al., 2021). Neuroimaging studies suggest that chronic stress influences motivation-related brain function in an inverted U-shaped manner, including activity in regions such as the prefrontal cortex, hippocampus, and amygdala (Friedman and Robbins, 2022; Maggio and Segal, 2019; B. S. McEwen et al., 2015; McEwen et al., 2016). Additionally, chronic stress could impact dopamine secretion and excitability of dopaminergic receptors in an inverted U-shaped manner in humans (Baik, 2020; Douma and de Kloet, 2020; Marinelli, 2007; Sandi, 2013; Stanwood, 2019). According to the allostatic load and reactive scope models (Gangloff and Greenberg, 2023; McEwen, 1998; Guidi et al., 2020; Romero et al., 2009), within a low-to-moderate range of stress, chronic stress increases various physiological mediators (e.g., heart rate, corticosterone) to trigger coping responses, but excessively high levels of stress can result in dysfunction. In other words, a moderate level of stress may optimize performance. Previous studies have shown that chronic stress can affect the activation of motivation-related brain regions and dopamine levels, but limited human studies have investigated the relationship between chronic stress and motivation, as well as the underlying mechanisms.

To investigate the relationship between chronic stress and the motivation to expend effort for rewards, we measured participants' chronic stress levels following a major life event and their decision-making preferences in an effort-reward task. Following previous studies, (Duan et al., 2015; Gao et al., 2022; Zhang et al., 2016), undergraduates preparing for the National Postgraduate Entrance Examination (NPEE), one of the most important and highly competitive exams within the Chinese educational system, participated in the study. We adopted an effort-reward task from previous studies Gheza et al. (2018), wherein participants could decide whether to complete an extra dot-click task for rewards or not. Participants with higher motivation to obtain rewards could choose to complete the extra dot-click task more frequently despite of effort cost. In other words, a higher acceptance rate indicates that participants are more willing to allocate effort to obtain rewards (Gheza et al., 2018; Gheza et al., 2023). Previous studies showed that dopamine could increase individuals' willingness to accept high-effort offers to obtain rewards (Bogdanov et al., 2022; McGuigan et al., 2019; Westbrook et al., 2020). The Drift Diffusion Model (DDM), a widely-used approach to capture individuals' preferences in reward decisions, was used to decompose participants' decision making (Dutilh and Rieskamp, 2016; Harris and Hutcherson, 2022; Ratcliff and McKoon, 2008). The drift rate (v), a parameter indicating the average rate of evidence accumulation (Ratcliff and McKoon, 2008; Ratcliff et al., 2016), was proved to be positively correlated with participants’ motivation for rewards (Calabro et al., 2023; Dutilh and Rieskamp, 2016; Lee and Usher, 2023; Y. Liu and Lourenco, 2022). Furthermore, considerable empirical evidence has shown that the drift rate can be modulated by dopamine, and dopamie increases the drift rate in the presence of potential rewards (Pagnier et al., 2024; Westbrook et al., 2020).

Additionally, we evaluated the reward positivity (RewP), an ERP component that typically peaks approximately 200–300 ms following feedback onset over frontal-central electrodes (Glazer et al., 2018; Proudfit, 2015; Webber et al., 2024). Converging evidence suggests that its amplitude reflects reward responsiveness and motivational salience (Bress and Hajcak, 2013; Glazer et al., 2018; Proudfit, 2015; Yan et al., 2023) and shows a positive association with reward motivation (Bowyer et al., 2021; Pegg and Kujawa, 2020; Threadgill and Gable, 2016; Yeung et al., 2005). Furthermore, RewP originates in the ACC and VS (Kiat et al., 2016; Proudfit, 2015), and its amplitude scales with phasic release of dopamine in the mesolimbic dopamine system (Foti et al., 2015; Foti et al., 2011; Holroyd and Coles, 2002). Therefore, RewP serves as an ideal ERP component for measuring the motivation to expend effort for rewards and the activation of the mesolimbic dopamine system. Although limited research has investigated the relationship between chronic stress and motivation, empirical evidence showed that chronic stress has a U-shaped relationship with RewP (Yi et al., 2024).

The allostatic load and reactive scope models suggest an inverted U-shaped relationship between stress levels and mental functions, and empirical evidence supports a similar relationship between stress and dopamine levels. Based on these findings, we hypothesized that chronic stress would exhibit an inverted U-shaped relationship with motivation. Specifically, we predicted that participants’ motivation to exert effort for reward would increase as chronic stress levels rose from low to moderate, but decrease when stress levels became high. Accordingly, we expected that PSS scores would show an inverted U-shaped relationship with: 1) the proportion of effort choice, 2) the drift rate, and 3) RewP amplitude.

## 2 Materials and methods

### 2.1 Participants

Ninety undergraduates from Shenzhen University, who were preparing for the National Postgraduate Entrance Examination (NPEE), were recruited for this study. We used G∗Power 3.1 to calculate the sample size that was needed in the current study, with a statistical power of 0.80, an effect size of 0.15, and a significance level of α = 0.05 for the effect of stress on accept rate, v, and RewP (Gerber et al., 2018; Huvermann et al., 2021; Rawls et al., 2021; van der Groen et al., 2018). It suggested that 77 participants were needed, and we finally recruited 90 participants in the current study to better ensure the experimental validity. All participants were right-handed with either normal or corrected-to-normal vision and reported no history of neurological or psychological conditions (e.g., major depressive disorder, anxiety, and psychiatry), current use of psychotropic drugs, drug abuse, or smoking. They visited our laboratory approximately 1–2 months before taking the NPEE. However, five participants withdrew halfway, resulting in a final sample size of 85 participants (37 females, age: 22.45 ± 0.66 years). Each participant received compensation and provided informed consent following the protocol approved by the Shenzhen University Institutional Review Board.

### 2.2 Procedure

Participants visited our laboratory and completed questionnaires, including the Perceived Stress Scale (PSS), the trait anxiety version of the State-Trait Anxiety Inventory (STAI-T), Self-rating Depression Scale (SDS), and Adolescent Daily School Life Events Scale (ADSLES). Additionally, they were required to report information about their exam preparation. Subsequently, they were seated comfortably before a computer screen and performed an effort-reward task adapted from Gheza et al. (2018).

### 2.3 The effort-reward task

**Fig. 1:**
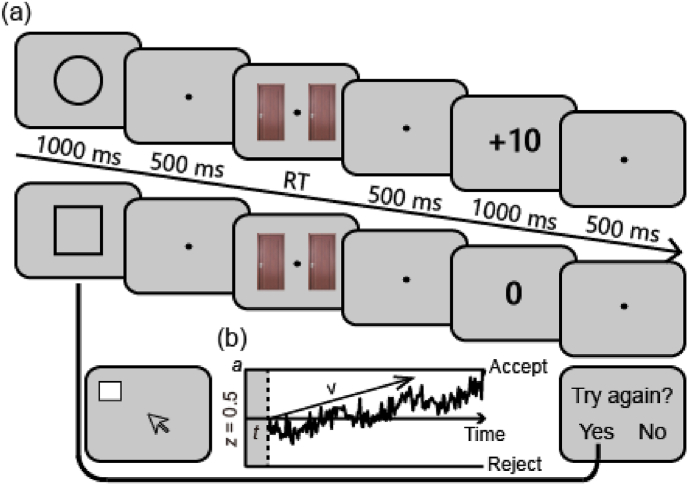
The effort-reward task and drift-diffusion model (DDM). (a) In the effort-reward task, participants have a 50 % probability of receiving either a reward delivery (+10) or feedback stimuli for reward omission (0) by choosing one of two doors in both regular and special trials. In special trials (indicated here starting with the square), if the reward was omitted, participants could choose to complete a dot-clicking task to earn an opportunity to choose again. (b) Representation of the drift-diffusion model and its parameters (starting point-*z*, drift rate-*v*, non-decision time-*t*, and boundary separation-*a*).

As depicted in Fig. 1, the effort-reward task consists of two conditions: regular and special trials. In both conditions, participants could receive a reward by choosing one of two doors. If they did not receive a reward during the special trials, they could earn another chance to make a choice by completing a random dot-clicking task.

At the start of each trial, a 1000-ms cue (square or circle, counterbalanced between subjects) appeared to inform participants whether the current trial was regular or special. After a fixation period lasting from 500 ms to 700 ms, two doors were displayed at the center of a computer screen. Participants are required to select a door behind which a potential reward (10 points) may be concealed. Following a 500-ms interval, a feedback stimulus appeared for 1000 ms to inform participants whether the reward was delivered or not (+10 or 0). However, the outcome of each trial was pre-determined, with an equal probability of 50 % for receiving a reward in both conditions. Additionally, in the special trial, participants had the opportunity to make an effort decision if the reward was omitted. They were required to decide whether to accept or reject an offer to complete a random dot-clicking task to earn another choice opportunity. If they accept the offer, a small square measuring 2.5 × 2.5 mm appears randomly on the computer screen. They were required to click on it using the mouse. The task consisted of 10 iterations, each with the square appearing at different locations. After completion of the random dot-clicking task, participants could get back to choose between two doors but the “reward door” was randomly allocated.

There was a total of 240 trials, divided into four blocks. Before the main experiment, each participant underwent a practice procedure consisting of eight trials to familiarize themselves with the task.

### 2.4 Questionnaires

The PSS is a 10-item self-report scale that is a reliable and valid tool for assessing chronic perceived stress (Cohen, 1988). Participants were required to rate how frequently they had experienced each stress-related item over the previous month, ranging from 1 (never) to 5 (always). A higher score on the scale reflects an increased level of perceived stress over the past month.

To assess participants' health status and avoid any influence on the results, the STAI-T, SDS, and ADSLES were used. The STAI-T is a valid and reliable index of trait anxiety (Spielberger et al., 1983), which consists of 20 items assessing how respondents feel about each statement on the questionnaire in the past week (ranging from 1: not at all, and 4: very much). The SDS consists of 20 items that assess the frequency of experiences with each statement in the past week (ranging from 1: never to 4: always) (X. Liu et al., 1994; Zung, 1965). The raw sum score is converted to a 100-point scale for better comparison and analysis.

To assess various daily school life events experienced by participants, the Adolescent Self-Rating Life Events Checklist (ADSLES) was employed. The ADSLES is widely used to evaluate the frequency and intensity of stressful life events in adolescents (Xianchen Liu et al., 1997; Ou, 2021). It comprises 20 items with 1–5 points for each item. A higher score indicates a greater number of life-stress events.

### 2.5 EEG recording and preprocessing

A 32-channel wireless EEG acquisition system (Neuroscan Greal EEG, Australia) was used to record electroencephalogram (EEG) signals, with electrode arrangement configuration based on the international 10–20 system. The signals were amplified via DC mode and sampled at an online rate of 1024 Hz. A low-pass filter with a cutoff frequency of 100 Hz was applied, and the reference electrode was placed between FCz and Cz. Additionally, the resistance of all electrodes was kept below 5 kΩ throughout the task.

The EEG data were subjected to offline preprocessing using MATLAB R2021b (MathWorks, Natick, Ma, USA) and EEGLAB 2022.0 (Delorme and Makeig, 2004). All the data were resampled to a frequency of 500 Hz and re-referenced to the average value of bilateral mastoids. Then, these data were filtered with a band-pass filter ranging from 0.1 Hz to 30 Hz and then segmented into −200 ms–1000 ms relative to stimulus onset with a baseline correction from −200 to 0 ms relative to the feedback stimuli onset. Moreover, manual artifact selection and deletion were conducted, coupled with the utilization of an independent component analysis (ICA) algorithm for eye blink detection. A procedure was implemented to exclude additional epochs based on the following criteria: (1) a voltage difference exceeding 50 μV between adjacent sample points; (2) a voltage difference exceeding 200 μV or a maximum voltage difference smaller than 0.5 μV within 100-ms intervals. Finally, the cleaned epochs were individually averaged across conditions.

By integrating prior studies (Foti et al., 2011; Proudfit, 2015), the grand average waveform and topographical maps across different conditions in the current study, we identified the average amplitudes of the RewP between 220 and 280 ms following the onset of the feedback stimuli at the central-frontal area (FCz).

### 2.6 The DDM analysis

To further capture participants' willingness to allocate effort for reward, we conducted a DDM to fit trial-level RT and response data on effort decision using the HDDM toolbox (Wiecki et al., 2013). Following previous studies, we set the starting point (*z*) at 0.5 for a better fit to our data (Mandali et al., 2021). As depicted in Fig. 1, we fitted a model with following DDM parameters: 1) the drift rate (*v*), which reflects the average rate of evidence accumulation during the task; 2) the non-decision time (*t*), which indicates the duration of preparatory processes and the post-decision phase; 3) the boundary separation (*a*), which defines how much evidence is integrated before a response is initiated (Ratcliff and McKoon, 2008; Wiecki et al., 2013). We modeled the Bayesian posterior distribution of these parameters via Markov chain Monte Carlo (MCMC) sampling. Additionally, we sampled 10,000 observations from the posterior distribution, excluding the initial 5000 "burn-in" samples. Trace and autocorrelation plots were examined to evaluate convergence. To assess the model's quality, the estimated parameters were employed in generating simulated data through posterior predictive checks.

### 2.7 Data analysis

Statistical analyses were implemented in IBM SPSS Statistics 26.0. Initially, we analyzed the behavioral data, subjective ratings, and ERP components to confirm the effectiveness of reward manipulation. We conducted paired samples t-tests to analyze the response time (RT) between trial types, as well as the offer acceptance rate, drift rate, and ΔRewP (the difference in the difference wave of reward delivery and omission in special and regular trials) between males and females. For the RewP, a trial type (regular, special) × valance (reward omission, reward delivery) ANOVA was conducted.

To elucidate the relationships among different variables, we conducted correlation analyses among biological sex, STAI-T, SDS, ADSLES, offer acceptance rate, drift rate, and ΔRewP.

To investigate the relationship between chronic academic stress and the motivation to expend effort to obtain rewards, we conducted hierarchical regression analyses. In model 1, the linear model (Y = *β*_0_ + *β*_1_x) was initially included, followed by the quadratic model (Y = *β*_0_ + *β*_1_x + *β*_2_x^2^) in model 2 (where Y indicates the dependent variable, *β*_0_ represents the constant, *β*n denotes the regression coefficient and x indicates the independent variable). Additionally, correlation analyses revealed significant or marginally significant correlations between biological sex and the dependent variables. Therefore, biological sex was introduced as a control variable in Model 1 to account for its potential influence. In Model 2, the linear model was included, and in Model 3, the quadratic model was included. The dependent variables consisted of (1) the offer acceptance rate and drift rate per subject and (2) ΔRewP.

## 3 Results

### 3.1 Descriptive data

Participants reported that, on average, they prepared for the NPEE for 5.76 ± 2.22 months and spent 7.82 ± 2.11 h per day studying for the exam. Additionally, the average PSS score was 27.93 (*SD* = 6.52). Table 1 provides a detailed overview of the means and standard deviations of the questionnaire scores as well as correlations between questionnaire scores and measures of offer acceptance rate, drift rate, and ΔRewP. Notably, participants' biological sex demonstrated significant correlations with both the offer acceptance rate and drift rate (*p*s < 0.05). The paired samples t-tests revealed that males showed higher offer acceptance rates compared to females (0.80 *vs.* 0.60, *p* = 0.001), as well as higher drift rates (1.42 *vs.* 0.55, *p* = 0.003). While the correlation between biological sex and ΔRewP did not reach statistical significance, a marginal correlation was observed (*r* = −0.19, *p* = 0.085). The paired samples *t*-test revealed that there was a marginally significant main effect of biological sex on the ΔRewP (*t*(83) = 1.74, *p* = 0.085, Cohen'*d* = 0.38), which was higher in males (*M* = 1.43, *SE* = 0.50) relative to females (*M* = 0.16, *SE* = 0.52).

**Table 1:** Means, standard deviations, and intercorrelations among measures for all subjects.

Variables	*M*	*SD*	1	2	3	4	5	6	7	8
1. Sex	–	–	–							
2. PSS	27.93	6.52	0.42^b^	–						
3. ADSLES	8.86	6.18	0.10	0.29^b^	–					
4. STAI-T	44.96	10.48	0.32^b^	0.87^b^	0.34^b^	–				
5. SDS	49.33	10.01	0.22^a^	0.79^b^	0.34^b^	0.86^b^	–			
6. Effort acceptance	0.71	0.30	−0.34^b^	−0.19	−0.16	−0.18	−0.12	–		
7. Drift rate	1.04	1.37	−0.32^b^	−0.21	−0.16	−0.19	−0.14	0.95^b^	–	
8. ΔRewP	0.87	3.38	−0.19	0.09	0.11	0.03	−0.06	0.13	0.15	–

a *p* < *0.05*.

b *p* < *0.01; sex: 0 = male,1 = female*.

### 3.2 Behavioral results

#### 3.2.1 Reward choice

For participants' response times when choosing a door, there was a significant main effect of trial type, *t*(84) = 4.154, *p* < 0.001, Cohen'*d* = 0.91. Specifically, participants responded more rapidly in regular trials (*M* = 392.73 ms, *SE* = 21.00) compared to special trials (*M* = 425.07 ms, *SE* = 23.58).

#### 3.2.2 Effort decision and DDM results

On average, the offer acceptance rate was 71.08 % (±29.64 %) and participants spent 860 ms (±356 ms) to make this decision. To further capture the process of effort decision, we conducted a DDM for participants’ responses and RTs, the mean drift rate was 1.04 (±1.37). Moreover, there was a significant correlation between drift rate and effort acceptance rate (*r* = 0.95, *p* < 0.001).

#### 3.2.3 Relationship between PSS scores and effort decision

**Fig. 2:**
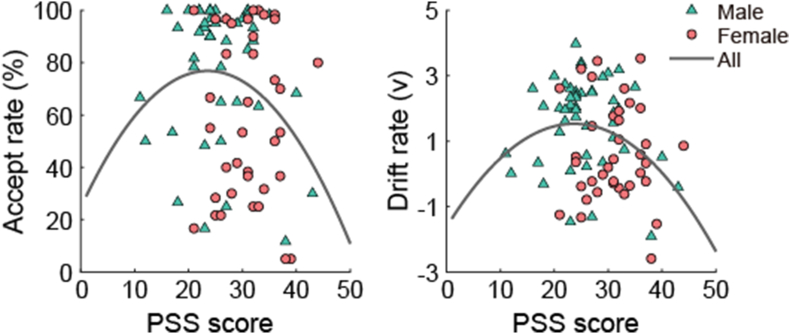
Scatterplots depict the associations between PSS score and the offer acceptance rate and drift rate. These plots may reveal curvilinear patterns within the entire sample.

As shown in Table 1, we did not find significant linear correlations between the PSS scores and the offer acceptance rate (*r* = −0.19, p = 0.084), as well as the drift rate (*r* = −0.21, p = 0.056). Fig. 2 shows the scatterplots of these data. To explore these relationships further, hierarchical regression analyses were performed with effort accept rate and drift rate as dependent variables. In model 1, we included the PSS scores to investigate the linear relationship between perceived stress and the behavioral measures of motivation to exert effort for rewards. In model 2, the square of the PSS scores was included to examine the curvilinear relationship between chronic stress and the behavioral measures of motivation. As shown in Table 2, results show a significant curvilinear relationship was observed between the PSS and the drift rate (β = −1.47, *p* = 0.028), explaining 6.0 % of the variance. Also, the results revealed marginally significant linear correlations with the offer acceptance rate (β = −0.19, *p* = 0.084) and drift rate (β = −0.21, *p* = 0.056). Additionally, a marginally significant curvilinear relationship was found with the offer acceptance rate (β = −1.18, *p* = 0.080).

**Table 2:** Hierarchical regression analysis (with and without sex as covariate) with PSS scores as a predictor and each behavioral outcome as the dependent variable.

**Independent Variables**		**Dependent Variables**									
		**Offer acceptance rate**					Drift rate				
		β	*t*	*p*	R^2^	ΔR^2^	β	*t*	*p*	R^2^	ΔR^2^
***without sex***											

*Model 1*					0.04	0.04				0.04	0.04
	PSS	−0.19	−1.75	0.084			−0.21	−1.94	0.056		
*Model 2*					0.07	0.04				0.10	0.06
	PSS	0.98	1.47	0.146			1.24	1.89	0.062		
	PSS^2^	−1.18	−1.77	0.080			−1.47	−2.24	0.028		

***with sex***											

*Model 1*					0.12	0.12				0.10	0.10
	Sex	−0.34	−3.32	0.001			−0.32	−3.06	0.003		
*Model 2*					0.12	0.00				0.11	0.01
	Sex	−0.32	−2.80	0.006			−0.28	−2.44	0.017		
	PSS	−0.05	−0.46	0.644			−0.09	−0.78	0.440		
*Model 3*					0.17	0.05				0.18	0.07
	Sex	−0.34	−3.04	0.003			−0.31	−2.74	0.007		
	PSS	1.30	2.018	0.047			1.53	2.39	0.019		
	PSS^2^	−1.36	−2.13	0.036			−1.63	−2.57	0.012		

*Notes:* Without Sex as a covariate, Model 1: Y = β0 + β1∗PSS + ϵ; Model 2: Y = β0 + β1∗PSS + β2∗PSS2 + ϵ; With Sex as a covariate, Model 1: Y = β0 + β1∗Sex + ϵ; Model 2: Y = β0 + β1∗Sex + β2∗PSS + ϵ; Model 3: Y = β0 + β1∗Sex + β2∗PSS + β3∗PSS2 + ϵ. Y indicates Dependent Variables (i.e., acceptance rate or drift rate); β0 indicates intercept, β1, β2, and β3 indicate regression coefficients, and ϵ indicates residual.

Given the observed correlations between biological sex and effort decision, we conducted a hierarchical regression analysis with the PSS as an independent variable and behavioral measures as dependent variables, controlling for biological sex. In model 1, biological sex was included as a control variable. Subsequently, the PSS score was included in model 2, and the square of the PSS score was included in model 3. The results showed no significant linear correlation between PSS and the behavioral measures of motivation (*p*s > 0.1). However, there was a significant curvilinear relationship between the PSS scores and the effort accept rate (β = −1.36, *p* = 0.036), as well as the drift rate (β = −1.63, *p* = 0.012). The quadratic model explained 5.0 % of the variance of effort accept rate and 7.0 % of the variance of drift rate respectively.

### 3.3 Electrophysiological data

#### 3.3.1 RewP

**Fig. 3:**
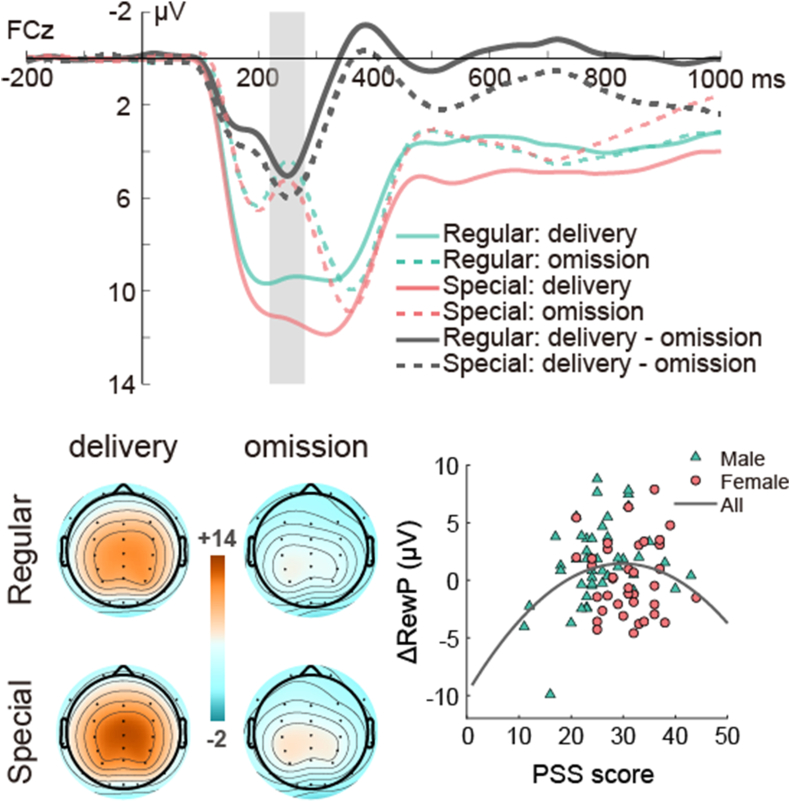
Average amplitude and scalp topography of RewP and the associations between PSS score and the ΔRewP. The shaded areas indicate the time window (220–280 ms) during which the mean RewP amplitude was measured. The scatter plot may reveal a curvilinear pattern between PSS scores and the ΔRewP within the entire sample.

As shown in Fig. 3, the RewP was elicited by reward feedback stimuli and reached its peak at 220–280 ms in central-frontal areas. Analyses revealed significant main effects of reward valance, *F*(1, 84) = 177.76, *p* < 0.001, η_p_^2^ = 0.68, and trial type, *F*(1, 84) = 32.71, *p* < 0.01, η_p_^2^ = 0.28. The amplitudes of RewP were enhanced in the reward delivery condition (*M* = 10.31 μV, *SE* = 0.68) compared to the reward omission condition (*M* = 4.26 μV, *SE* = 0.47), as well as in the special trials (*M* = 7.96 μV, *SE* = 0.57) relative to the regular trials (*M* = 6.62 μV, *SE* = 0.53). Furthermore, there was a significant interaction between trial types and reward valence, *F*(1, 84) = 5.70, *p* = 0.019, η_p_^2^ = 0.06. Post hoc analyses revealed that the difference of RewP between reward delivery and reward omission was more pronounced in special trials (11.20 μV *vs.* 4.71 μV, *p* < 0.001) compared with regular trials (9.42 μV *vs.* 3.81 μV, *p* < 0.001).

#### 3.3.2 Relationship between PSS scores and RewP

As illustrated in Fig. 3, the scatterplot suggested a potential quadratic relationship between the PSS scores and ΔRewP. Subsequently, hierarchical regression analyses were conducted to explore this relationship. We included the PSS in model 1 and the square of the PSS scores in model 2 to examine the linear and curvilinear relationship between chronic stress and the electrophysiological measures of motivation. As shown in Table 3, the results revealed no significant linear association between PSS and ΔRewP (β = 0.09, *p =* 0.439). However, a significant curvilinear relationship between the PSS and the ΔRewP was found (β = −1.40, *p* = 0.040), which explained 5.0 % of the variance of the ΔRewP.

**Table 3:** Hierarchical regression analysis (with and without sex as covariate) with PSS scores as a predictor and ΔRewP as the dependent variable.

**Independent Variables**		Dependent Variables				
		ΔRewP				
		*β*	*t*	*p*	R2	ΔR2
***without sex***						

*Model 1*					0.01	0.01
	PSS	0.09	0.78	0.439		
*Model 2*					0.06	0.05
	PSS	1.46	2.18	0.032		
	PSS^2^	−1.40	−2.08	0.040		

***with sex***						

*Model 1*					0.04	0.04
	Sex	−0.19	−1.74	0.085		
*Model 2*					0.07	0.03
	Sex	−0.27	−2.32	0.023		
	PSS	0.20	1.71	0.092		
*Model 3*					0.13	0.06
	Sex	−0.23	−2.60	0.011		
	PSS	1.74	2.65	0.010		
	PSS^2^	−1.55	−2.38	0.019		

***Notes:*** Time window: 220–280 ms. Site: FCz. Without Sex as a covariate, Model 1: Y = β0 + β1^a^PSS + ϵ; Model 2: Y = β0 + β1^a^PSS + β2^a^PSS2 + ϵ; With Sex as a covariate, Model 1: Y = β0 + β1^a^Sex + ϵ; Model 2: Y = β0 + β1^a^Sex + β2^a^PSS + ϵ; Model 3: Y = β0 + β1^a^Sex + β2^a^PSS + β3^a^PSS2 + ϵ. Where Y indicates Dependent Variables (i.e., ΔRewP); β0 indicates intercept, β1, β2, and β3 indicate regression coefficients, and ϵ indicates residual.

Moreover, we performed a hierarchical regression analysis with biological sex as a control variable in model 1. Subsequently, we included PSS in model 2 and the square of the PSS sores in model 3. Similarly, there was a significant curvilinear relationship (β = −1.55, *p* = 0.019) between PSS and the ΔRewP, but no significant linear correlation (β = 0.20, *p* = 0.092). The quadratic model explained 6.0 % of the variance of ΔRewP.

## 4 Discussion

The present study investigated the relationship between chronic stress and the motivation to expend effort for rewards using both behavioral and neurophysiological measures. Consistent with prior research, reward delivery elicited higher RewP waveforms compared with reward omission, which was particularly evident under special trials wherein an additional effort choice was available (Gheza et al., 2018). Crucially, there were inverted U-shaped relationships between the PSS scores and the offer acceptance rate, drift rate, and ΔRewP (i.e., the difference in the difference waves of reward delivery and omission in special and regular trials). These findings suggest that chronic stress impacts the motivation to allocate effort for rewards such that moderate stress increases, while high and low stress decreases, the motivation for reward.

As expected, at the behavioral level, there was a marginally significant quadratic relationship between the PSS scores and the offer acceptance rate, and the quadratic relationship reached significance when controlling for biological sex. Numerous studies have highlighted the importance of biological sex as a significant influencing factor in motivating effort for rewards (Omansky et al., 2016; Rosenfeld and Tomiyama, 2021; Soutschek et al., 2017). Additionally, the results revealed that males showed higher offer acceptance rate, drift rate, and ΔRewP than females. Additionally, our results revealed that males showed higher offer acceptance rate, drift rate, and ΔRewP than females. These results are consistent with previous studies showing that males are more likely to choose difficult tasks than females. Previous studies suggest that oxytocin can reduce motivations to exert effort for reward both in human and mice (Plessow et al., 2018; Wald et al., 2020). In the current study, females exhibited blunted motivations to allocate effort for rewards compared to males, which may be linked to higher oxytocin levels in females. Previous studies have revealed that participants who were more willing to expend effort for rewards were more likely to complete the additional task (Bogdanov et al., 2022; Gheza et al., 2018). Our results demonstrate an inverted U-shaped relationship between chronic stress and the motivation to allocate effort for rewards. That is, participants would not be willing to allocate effort when exposed to low or high levels of chronic stress but had a relatively higher motivation for allocating effort when exposed to a moderate level of chronic stress.

Furthermore, the quadratic association observed between the PSS scores and drift rate during effort decision making, regardless of biological sex, lends support to the notion of an inverted U-shaped relationship between chronic stress and motivation. The DDM has been widely used to decompose the process of decision making (Harris and Hutcherson, 2022; Ratcliff and McKoon, 2008). Drift rate, serving as a metric for the average rate of evidence accumulation, shows a positive correlation with motivation to expend effort (Eikemo et al., 2017; Leng et al., 2022; Y. Liu and Lourenco, 2022). Our results revealed a significant quadratic relationship between the PSS scores and drift rate, suggesting an inverted U-shaped relationship between chronic stress and the motivation to expend effort for rewards. Prior research has investigated the impacts of acute stress on participants' willingness to exert effort and found that acute stress tends to diminish their motivation (Bogdanov et al., 2021; Vriens, 2021), and also observed alterations of motivation influenced by chronic stress (Omansky et al., 2016; Schwabe et al., 2008). Our behavioral results extended these findings by including participants exposed to a major life event (i.e., the NPEE), revealing an inverted U-shaped relationship between chronic stress and motivation.

Similarly, at the electrophysiological level, there was a significant quadratic relationship between the PSS scores and the ΔRewP irrespective of biological sex. Consistent with previous studies, the RewP was enhanced when reward was delivered compared to when it was omitted (Proudfit, 2015; Yi et al., 2023), and this difference was more pronounced in special trials compared to regular trials (Gheza et al., 2018). Previous studies have found that RewP was enhanced towards reward delivery compared with reward omission (Foti et al., 2015; Proudfit, 2015; Threadgill and Gable, 2016), as well as in response to high relative to low magnitude rewards (Paul et al., 2020; Sambrook and Goslin, 2015; Yaple et al., 2018; Yi et al., 2023), suggesting that the amplitude of RewP reflects rewards responsiveness and motivational salience of outcome (Soder and Potts, 2018; Yeung et al., 2005). Additionally, the amplitudes of RewP scale with an individual's motivation to exert effort (Bowyer et al., 2021; Pegg and Kujawa, 2020; Threadgill and Gable, 2016; Yeung et al., 2005). Furthermore, converging evidence suggested that the amplitudes of RewP were associated with activations of the dopaminergic system (Brown et al., 2020). Our results revealed that the PSS scores had a significant quadratic relationship with ΔRewP, which may reflect the activations of the dopaminergic system when encountering potential rewards. Considering the associations between RewP and motivational salience, these results may imply that participants who were exposed to moderate levels of chronic stress may manifest higher motivation.

While existing human research on the relationship between chronic stress and reward motivation is limited, evidence from animal models suggested that chronic stress reduced effort-related behaviors (Cerniauskas et al., 2019; Kleen et al., 2006; Rygula et al., 2005; Yoshida et al., 2021). The results of these animal models differ from our findings in several key aspects. On the one hand, these animal studies primarily focused on chronic stress and its impact on motivation, whereas our research treats chronic stress as a continuous variable, allowing for a more detailed examination of the relationship between varying levels of chronic stress and motivation. On the other hand, the animal studies employed life-threatening stressors such as food deprivation, water deprivation, and restraint, which likely induced a stronger level of chronic stress, positioning the subjects in the descending arm of the inverted U-shaped curve.

Previous studies have indicated that chronic stress influences the activation and connectivity of brain regions in an inverted U-shaped manner, such as the PFC and amygdala (Holmes and Wellman, 2009; Bruce S. McEwen et al., 2016; Morey et al., 2016; Sandi, 2013). These regions are known to play crucial roles in motivating individuals to allocate effort for rewards. Consequently, chronic stress may exhibit an inverted U-shaped impact on motivation-related brain regions, leading to a corresponding relationship between chronic stress and the motivation to exert effort for rewards. Furthermore, converging evidence has suggested an inverted U-shaped impact of chronic stress on dopamine levels and the excitability of dopaminergic receptors (Douma and de Kloet, 2020; Ott and Nieder, 2019; Stanwood, 2019). Although our study did not directly manipulate the participants' dopamine levels, the amplitude of RewP is indicative of mesolimbic dopamine system activation, and its magnitude is positively associated with dopamine levels (Foti et al., 2011; Proudfit, 2015). Given that dopamine plays a crucial role in motivating individuals to allocate effort, our study indirectly supports the idea that chronic stress influences participants' dopamine levels in the brain, thereby affecting their motivation to exert effort for rewards in an inverted U-shaped manner.

Our study provides empirical support for the allostatic load and reactive scope models (McEwen, 1998; Guidi et al., 2020; Romero et al., 2009). The results revealed an inverted U-shaped relationship where motivation first rises with increasing chronic stress up to an optimal level, beyond which further stress leads to decline. Furthermore, these findings have significant implications for understanding behavioral patterns in individuals exposed to chronic stress and for comprehending the initiation and progression of mental disorders. Previous studies have observed that an individual's motivation to seek rewards is influenced by chronic stress levels; however, the specific pattern through which chronic stress affects motivation needs further investigation. Our study recruited participants who were experiencing a major life event (i.e., NPEE) and revealed an inverted U-shaped relationship between chronic stress and the motivation to exert effort for rewards. In the face of life and economic stressors, an increasing number of people are opting for a "lying flat" lifestyle, leading to a decrease in motivation for both learning and work (Hsu, 2022; Zheng et al., 2023). Our findings contribute to explaining the underlying mechanism for this phenomenon. It is evident that extremely low or high level of stress are detrimental to the motivation for working and learning. These findings provided insights into our daily life, suggesting that maintaining an optimal level of stress can enhance motivation in the pursuit of long-term goals. Moreover, dysfunction in the motivation to allocate effort was identified as a core symptom of chronic stress-induced mental disorders (Catalano and Green, 2023; Nawijn et al., 2015; Rohleder, 2016; Starcke and Brand, 2012). However, the relationship between chronic stress and the motivation to expend effort for rewards, as well as its mechanism, remains unknown. Our research addresses this gap in the literature, suggesting that impairments in the motivation to expend effort, observed with low or high levels of chronic stress, may represent a potential mechanism through which chronic stress contributes to associated disorders. In other words, a moderate level of stress may be beneficial for an individual's psychological resilience and overall well-being (Aschbacher et al., 2013; B. S. McEwen et al., 2015).

Nevertheless, this study has several limitations that warrant discussion. Firstly, although our study revealed an inverted U-shaped relationship between chronic stress and the motivation to allocate effort, we refrain from drawing causal conclusions. Ethical considerations prevented us from inducing chronic stress in participants within a laboratory setting to investigate its impact on motivation directly. Subsequent research is warranted to further elucidate how chronic stress influences the motivation to expend effort for rewards in humans, using alternative methodologies such as longitudinal design and cross-lagged analysis (Arjas and Parner, 2004; Farrington, 1991; Finkel, 1995). Secondly, the influence of biological sex on our results is noteworthy given sex differences in stress-related disorders such as depression. We did not, however, assess gender role orientation (Bem, 1974), which may interact with biological sex to influence the relationship between stress and mental health. Future research should address this interaction. Thirdly, as Gheza et al. (2018) mentioned, the effort cost of dot-clicking may be partially confounded by opportunity cost, as time is required to complete the clicking. However, previous studies have suggested that effort cost may stem, at least partially, from the time spent on the task as it progresses (Inzlicht et al., 2018, Inzlicht et al., 2018; Kurzban et al., 2013). Nevertheless, our research still suggests that participants were more motivated to obtain rewards when encountering moderate levels of chronic stress irrespective of effort or time costs in doing the extra dot-clicking task.

In conclusion, we investigated the relationship between chronic stress and the motivation to allocate effort for rewards by recruiting participants who were preparing for the NPEE. The results unveiled a quadratic correlation between PSS scores and the proportion of effort acceptance, drift rate, and ΔRewP. Our findings support the idea that chronic stress demonstrates an inverted U-shaped relationship with the motivation to exert effort for rewards, evident at both behavioral and electrophysiological levels. Notably, an individual's motivation to allocate effort for rewards tends to diminish significantly when exposed to extremely low or high levels of chronic stress in comparison to a moderate level. Our findings carry substantial implications for grasping the behavioral patterns exhibited by individuals under chronic stress and for gaining insights into the onset and maintenance of mental disorders.

## CRediT authorship contribution statement

**Wei Yi:** Writing – review & editing, Writing – original draft, Formal analysis, Conceptualization. **Xin Li:** Investigation, Formal analysis. **Wangxiao Chen:** Investigation, Formal analysis. **Linlin Yan:** Writing – review & editing, Methodology. **Fei Xin:** Writing – review & editing, Validation. **Tony W. Buchanan:** Writing – review & editing, Validation. **Jianhui Wu:** Writing – review & editing, Validation, Supervision.

## Declaration of competing interest

All authors report no potential financial conflicts of interest.

## Data Availability

Data will be made available on request.
